# Axillary staging in breast cancer patients treated with neoadjuvant chemotherapy in two Dutch phase III studies

**DOI:** 10.18632/oncotarget.15101

**Published:** 2017-02-04

**Authors:** Birgit E.P. Vriens, Kristien B.M. Keymeulen, Judith R. Kroep, Ayoub Charehbili, Petronella G. Peer, Maaike de Boer, Maureen J.B. Aarts, Esther M. Heuts, Vivianne C.G. Tjan-Heijnen, The Dutch Breast Cancer Research Group (BOOG)

**Affiliations:** ^1^ Department of Medical Oncology, GROW-School for Oncology and Developmental Biology, Maastricht University Medical Center, Maastricht, The Netherlands; ^2^ Department of Surgery, GROW-School for Oncology and Developmental Biology, Maastricht University Medical Center, Maastricht, The Netherlands; ^3^ Department of Medical Oncology, Leiden University Medical Center, Leiden, The Netherlands; ^4^ Department of Surgery, Leiden University Medical Center, Nijmegen, The Netherlands; ^5^ Biostatistics, Radboud Institute for Health Sciences, Nijmegen, The Netherlands

**Keywords:** breast cancer, neoadjuvant chemotherapy, sentinel node procedure, node-negative

## Abstract

**Background:**

Primary aim of our study was to assess the impact of timing of sentinel node procedure, pre- versus post-neoadjuvant chemotherapy, on final pathologic node-negative rate (pN0) in patients with clinically node-negative (cN0) breast cancer. Secondary endpoint was the usability of the sentinel node procedure in patients with clinically node-positive disease that converted to cN0 after neoadjuvant chemotherapy.

**Patients and Methods:**

Patients were enrolled in two sequentially conducted Dutch phase III trials, studying the impact of two neoadjuvant chemotherapy schedules and use of zoledronic acid on complete pathologic response rate. For the present analyses, patients were excluded if they had not undergone surgical axillary staging.

**Results:**

In total 439 patients were included, of whom 230 (52%) had pre-treatment cN0. In this group, pN0 status was seen in 58% (*N* = 23) of patients with a sentinel node biopsy post-neoadjuvant chemotherapy compared to 51% (*N* = 83) pre-neoadjuvant chemotherapy, including the axillary lymph node dissection whenever performed. In multivariable analysis, timing of sentinel node procedure (pre- versus post- neoadjuvant chemotherapy) was, however, not significantly associated with final pN0/pN0(i+) status, with an odds ratio of 1.18 (95% CI 0.64 - 2.18) after correction for age, clinical tumor status, histology, grade, hormone- and HER2 receptor. Of patients with clinically node-positive disease only 15% had a final pN0 status, with a false-negative rate of the sentinel node of 30%.

**Conclusion:**

In breast cancer patients with cN0 disease, sentinel node procedure performed post-neoadjuvant chemotherapy led to nodal down staging, although not statistically significant after multivariate correction for patient and tumor characteristics.

## INTRODUCTION

Neoadjuvant chemotherapy in breast cancer patients provides the opportunity to monitor treatment effects *in vivo*, which may also have a positive effect on coping with the disease for the patient, and has the potential to downstage the primary tumor which may facilitate breast conserving surgery. Neoadjuvant chemotherapy has also shown to eradicate nodal disease in 20% to 40% of patients [1–3]. Obviously, if the axillary lymph nodes are truly negative, there can be no possible benefit from performing an axillary lymph node dissection. This raises the question whether the current widespread policy of performing a sentinel node procedure at initial diagnosis *pre*-neoadjuvant chemotherapy in patients with at baseline clinical node-negative disease is still appropriate. Another question is whether performing a sentinel node procedure *post-*neoadjuvant chemotherapy may potentiate axilla-conserving treatment in patients with clinically node-positive disease at initial diagnosis when converted to clinical node-negative disease.

Previously, we reported a systematic review on twenty-seven studies including 2148 patients with regard to the accuracy of sentinel node biopsy post-neoadjuvant chemotherapy [4]. The majority of the studies included both clinically node-negative and clinically node-positive patients. The pooled sentinel node identification rate, false negative rate (FNR), negative predictive value and accuracy were 90.9%, 10.5%, 89.0% and 94.4%, respectively. The reported sentinel node success rates were heterogeneous and several variables, amongst others clinical node-positivity pre-neoadjuvant chemotherapy, were reported to be associated with decreased sentinel node accuracy.

In two, quite recent, large sentinel node studies, the SENTINA study and the ACOSOG Z1071 study, it was confirmed that the sentinel node procedure post-neoadjuvant chemotherapy in patients with initially node-positive disease converted to clinically node-negative disease resulted in a lower sentinel node detection rate and higher false-negative rate compared to upfront sentinel node procedure performed in patients who had clinically node-negative disease at initial diagnosis [5, 6]. Currently, in patients with clinically node-negative disease, the timing of sentinel node procedure is still a matter of debate.

Considering the above, we conclude that the most optimal timing of sentinel node procedure in patients with *clinically node-negative disease (cN0) at diagnosis* who will undergo neoadjuvant systemic therapy still remains to be elucidated. Especially patients with nodal low-volume disease may achieve a pathologic complete remission in the lymph nodes. Therefore, we re-analyzed the data from two Dutch randomized phase III breast cancer studies on neoadjuvant chemotherapy with regard to final pathologic nodal status related to baseline clinical lymph node status and related to timing of axillary staging [1, 7].

## RESULTS

### Patient inclusion

**Figure 1:**
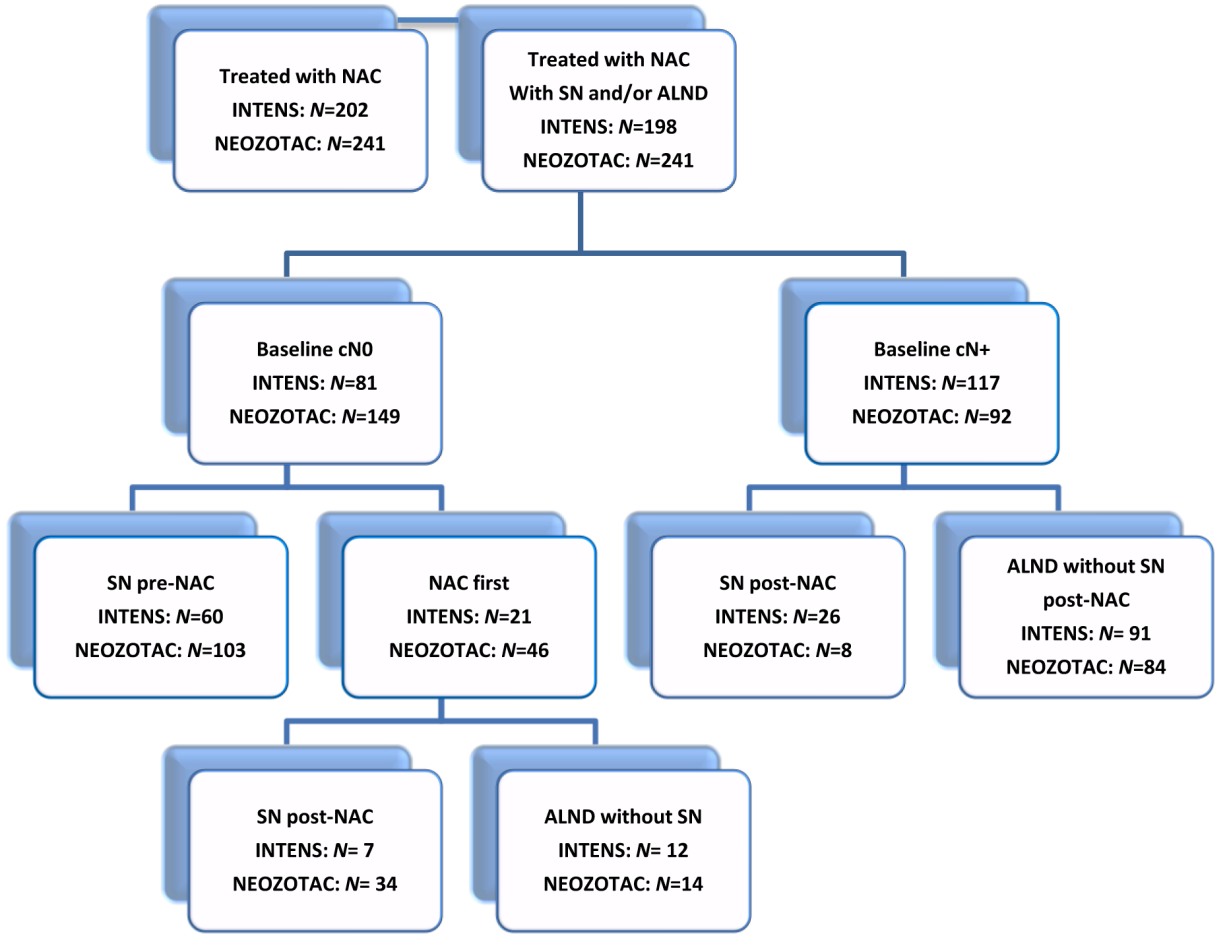
Consort diagram of axillary staging during neo-adjuvant chemotherapy in two Dutch phase III studies, the INTENS and NEOZOTAC studies Abbreviations: SN, sentinel node; ALND, axillary lymph node dissection; NAC, neo-adjuvant chemotherapy; Baseline cN0 based on ultrasound plus or minus.

Between February 2006 and May 2012, a total of 448 patients were enrolled in the Dutch INTENS (*N* = 202) and NEOZOTAC (*N* = 246) phase III trials. Of these, 9 patients did not undergo a sentinel lymph node procedure or axillary lymph node dissection and were, therefore, excluded. Of the 439 included patients, 230 (52%) had pre-treatment clinically node-negative disease and 209 (48%) node-positive disease, based on physical and ultrasound examination with or without cytology (Figure 1).

Of patients with at baseline clinically node-negative disease, a sentinel node procedure pre-neoadjuvant chemotherapy was done in 163 patients (71%) and axillary staging post-neoadjuvant chemotherapy in 67 patients (29%). Patients with a sentinel node pre-neoadjuvant treatment had a smaller tumor and more often of ductal histology compared to those with axillary staging with or without sentinel node post-neoadjuvant chemotherapy, but a larger tumor of lower grade when compared to those who underwent a sentinel node procedure post-neoadjuvant chemotherapy (Table 1).

**Table 1:** Characteristics of patients who underwent a sentinel node procedure pre-neoadjuvant chemotherapy or who first received neoadjuvant chemotherapy before axillary staging, categorized by baseline clinical nodal stage

			Baseline cN0				Baseline cN+		
			SN pre-NAC	NAC first			NAC first		
				Total	SN post-NAC	ALND without SN	Total	SN post-NAC	ALND without SN
			*N* = 163 (%)	*N* = 67 (%)	*N* = 41 (%)	*N* = 26 (%)	*N* = 209 (%)	*N* = 34 (%)	*N* = 175 (%)
Age (years)									
	≤ 50		97 (60)	41 (61)	23 (56)	18 (69)	127 (61)	20 (59)	107 (61)
	> 50		66 (40)	26 (39)	18 (44)	8 (31)	82 (39)	14 (41)	68 (39)
	Median (range)		48 (29 -68 )	49 (33 – 65)	50 (33 - 65)	49 (34 – 62)	49 (24 – 70)	49 (36 – 70)	49 (24 – 70)
cT-status									
	cT1-2		106 (65)	36 (54)	30 (73)	6 (23)	99 (47)	25 (74)	74 (42)
	cT3-4		57 (35)	31 (46)	11 (27)	20 (77)	110 (53)	9 (26)	101 (58)
Histology									
	Ductal		121 (77)	43 (68)	30 (73)	13 (57)	171 (86)	28 (88)	143 (86)
	Lobular		26 (16)	16 (25)	8 (20)	8 (35)	16 (8)	3 (9)	13 (8)
	Other		11 (7)	5 (8)	3 (7)	2 (9)	11 (6)	1 (3)	10 (6)
	Unknown		5	3	0	3	11	2	9
Histological grade									
	Grade I		30 (26)	10 (25)	5 (24)	5 (26)	19 (12)	3 (10)	16 (12)
	Grade II		66 (58)	21 (53)	10 (48)	11 (58)	76 (48)	14 (48)	62 (48)
	Grade III		18 (16)	9 (22)	6 (29)	3 (16)	63 (40)	12 (41)	51 (40)
	Unknown		49	27	20	7	51	5	46
Hormone receptor status									
	Positive		131 (80)	53 (79)	33 (80)	20 (77)	147 (70)	26 (76)	121 (69)
	Negative		32 (20)	14 (21)	8 (20)	6 (23)	62 (30)	8 (24)	54 (31)
HER2 status									
	Positive		11 (7)	2 (3)	1 (2)	1 (4)	31 (15)	10 (29)	21 (12)
	Negative		152 (93)	65 (97)	40 (98)	25 (96)	178 (85)	24 (71)	154 (88)
Hormone / HER2 status									
		Both negative	29 (18)	13 (19)	7 (17)	6 (23)	47 (22)	4 (12)	43 (25)
Study									
		INTENS	60 (37)	21 (31)	7 (17)	14 (54)	117 (56)	26 (76)	91 (52)
		NEOZOTAC	103 (63)	46 (69)	34 (83)	12 (46)	92 (44)	8 (24)	84 (48)
Axillary treatment									
		ALND	73 (45)	39 (58)	13 (32)	26 (100)	207 (99)	32 (94)	175 (100)

Abbreviations: SN, sentinel node; NAC, neoadjuvant chemotherapy; ALND, axillary lymph node dissection; Baseline cN0 and cN+ based on ultrasound plus or minus fine needle aspiration; Patients who underwent both SN pre-NAC and post-NAC were classified under SN pre-NAC

As could be expected, patients with clinically node-positive disease had more unfavorable primary tumor characteristics compared to patients with clinical node-negative breast cancer (Table 1). In patients with clinically node-positive disease, the tumors were larger, more often of ductal histology, grade III, ER negative and/or HER2 positive.

### Baseline node-negative

Of all patients with at baseline clinically node-negative disease, 52% had a (final) pathologic node-negative status, including the axillary lymph node dissection whenever performed (Table 2). Compared to patients with a sentinel node procedure pre-neoadjuvant chemotherapy, those with a sentinel node procedure post-neoadjuvant chemotherapy had more often a pathologic node-negative status, 58% (*N* = 23) *versus* 51% (*N* = 83) including the axillary lymph node dissection and 59% (*N* = 23) *versus* 53% (*N* = 86) based on sentinel node procedure alone (Table 2). A (final) pathologic node-negative status was seen in 56% (5/9) of patients with hormone receptor positive / HER2 positive breast cancer, in 48% (84/175) of cases with a hormone receptor positive / HER2 negative tumor, in 0% (0/4) of cases with a hormone receptor negative / HER2 positive tumor and in 74% (31/42) of cases with a hormone receptor negative / HER2 negative (triple negative) breast tumor.

**Table 2:** Primary and secondary endpoints of patients who underwent a sentinel node procedure pre-neoadjuvant chemotherapy or who first received neoadjuvant chemotherapy before axillary staging, categorized by baseline clinical nodal stage

		Baseline cN0				Baseline cN+		
		SN pre-NAC	NAC first			NAC first		
			Total	SN post-NAC	ALND without SN	Total	SN post-NAC	ALND without SN
		*N* = 163 (%)	*N* = 67(%)	*N* = 41 (%)	*N* = 26 (%)	*N* = 209(%)	*N* = 34 (%)	*N* = 175 (%)
Pathological nodal status (SN)								
	pN0(i-)/pN0(i+)	86 (53)		23 (59)			13 (39)	
	pN1mi	20 (12)		3 (8)			7 (21)	
	pNmacro	57 (35)		13 (33)			13 (39)	
	uk	0		2			1	
Pathological nodal status (SN + ALND)								
	pN0(i-)/pN0(i+)	83 (51)	37 (56)	23 (58)	14 (54)	48 (23)	5 (15)	43 (25)
	pN1mi	19 (12)	5 (8)	3 (7)	2 (8)	30 (14)	6 (18)	24 (14)
	pN1macro	61 (37)	24 (36)	14 (35)	10 (38)	129 (62)	22 (65)	107 (61)
	uk	0	1	1	0	2	1	1

Abbreviations: SN, sentinel node; ALND, axillary lymph node dissection; NAC, neoadjuvant chemotherapy; Baseline cN0 and cN+ based on ultrasound plus or minus fine needle aspiration; Patients who underwent both SN pre-NAC and post-NAC were classified under SN pre-NAC; uk, unknown.

Timing of sentinel node procedure (post- *versus* pre-neoadjuvant chemotherapy) was not significantly associated with final pN0/pN0(i+) status with an OR of 1.18 (95% CI 0.64 - 2.18) after correction for age, clinical tumor status, histology, histological grade, hormone receptor and HER2 status (Table 3).

**Table 3:** Impact of timing of axillary stage pre- versus post-neoadjuvant chemotherapy in patients with clinical node-negative disease that underwent a sentinel node procedure on final pN0/pN0(i+) status

		OR	95% CI	*N*
Unadjusted OR		1.23	0.69 – 2.19	229
Adjusted OR for 1 covariable	Age (≤ 50y/>50y)	1.25	0.70 – 2.22	229
	cT –status (cT1-2/cT3-4)	1.26	0.70 – 2.24	229
	Histology (ductal/lobular/other)	1.17	0.64 – 2.13	221
	Grade (G1/G2/G3)	1.28	0.61 – 2.69	154
	Hormone receptor (positive/negative)	1.22	0.68 – 2.18	229
	HER2-status (positive/negative)	1.20	0.68 – 2.15	229
Adjusted OR for all covariables		1.18	0.64 – 2.18	154

Unadjusted odds ratio (OR), OR adjusted for one of the covariates age, cT status, histology, tumor grade, hormone receptor status or HER 2 status and the adjusted OR for all previously mentioned covariates.

Of the 77 patients with sentinel node negative disease who did not undergo completion axillary lymph node dissection, 28 were included in the INTENS study. With a median follow up of 6 years three patients had distance recurrence and none had a regional recurrence. In the NEOZOTAC trial, follow-up duration is still too short to assess 5-year recurrence or survival rates.

### Baseline node-positive

Of patients with initially node-positive disease and a sentinel node procedure post-neoadjuvant chemotherapy because of a complete remission based on imaging evaluation (*N* = 34), 39% had a negative sentinel node. In all but one a complete axillary lymph node dissection was done. When including the results of axillary lymph node dissection, only 15% could still be classified as pathologic node-negative (Table 2).

For patients with initially node-positive disease who had a sentinel node procedure and completion axillary lymph node dissection post-neoadjuvant chemotherapy (*N* = 32), the false-negative rate of the sentinel node procedure was 30%.

Of all patients with initially node-positive disease, 23% had a pathologic node-negative status (Table 2).

## DISCUSSION

We performed a combined analysis on two sequentially conducted phase III trials using neoadjuvant chemotherapy in The Netherlands in the years 2006-2012. The main results of the two studies on impact of type of chemotherapy and role of zoledronic acid as an adjunct to neoadjuvant chemotherapy on pathologic complete remission rate in the breast and/or breast and lymph nodes have been reported before [1, 7].In the two studies, the timing of axillary staging was largely depended on the baseline clinical nodal status, although in patients with clinical node-negative disease the sentinel node procedure was increasingly used post-neoadjuvant chemotherapy in later years. Our findings show that axillary lymph node dissection post-neoadjuvant chemotherapy, compared to axillary staging pre-neoadjuvant chemotherapy, resulted more frequently in a final pathologic node-negative status (58% *versus* 51%) in patients with at baseline clinical node-negative disease, although this difference remained not statistically significant different after correction for age, clinical tumor status, histology, histological grade, hormone receptor and/or HER2 status. The absence of regional recurrences after a median follow-up of six years in those who did not undergo completion axillary lymph node dissection underscores the conclusion that performing the sentinel node procedure post-chemotherapy is safe in patients with clinically node-negative breast cancer at initial diagnosis.

The optimal timing of the sentinel node procedure with respect to neoadjuvant chemotherapy has almost exclusively been studied in patients who had initially clinical node-positive disease. Various meta-analyses, the recent prospective SENTINA cohort study and the ACOSOG Z1071 study have all shown a high false-negative rate in this particular subgroup of patients [5, 6]. In the SENTINA trial a false negative rate of 14.2% was reported and 24% if only 1 sentinel node was removed. ACOSOG Z1071 reported a false negative rate of 12.6% (in 81% of the patients two or more sentinel lymph nodes were removed). In our study, we also observed a high false-negative rate of 30% in patients with initially node-positive disease that converted to clinical node-negative disease. In 48% of these patients two or more sentinel nodes were removed. In the studies reported so far, long-term outcome of patients who converted from clinical-positive to sentinel node-negative without completion axillary lymph node dissection is not available. Especially for patients with triple negative disease who currently have no additional systemic therapy options outside a clinical trial setting after use of neoadjuvant chemotherapy, one can imagine that there might be an increased risk for regional recurrence if positive non-sentinel nodes are left behind. In our study, four patients with triple negative breast cancer converted from clinical positive to negative and underwent both a sentinel node procedure and completion axillary lymph node dissection. Of these, two had a positive sentinel node, and two had a negative sentinel node with no non-sentinel node metastases (i.e., no false-negative sentinel nodes).

Nevertheless, to be able to further de-escalate axillary treatment we should think of new ways to identify the nodal status of patients and to use new techniques or combination of existing techniques. It is well-known, that the reliability of the sentinel node procedure is enhanced by using both radiolabeled colloid and methylene blue (false-negative rate 10.8%) and by removing a higher number of nodes (false negative rate 9.1%) [5, 6, 8]. An alternative method is the use of a marker, e.g. radioactive iodine seed, selectively placed into the clinically most suspected positive-node [9]. In the study of Donker *et al*. the identification rate of the marked node with a radioactive iodine seed was 97% with a false negative rate of 7% [9]. In 5 of 30 patients with a negative marked node additional positive lymph nodes were found (negative predictive value of 83% (95% CI 65% - 94%)). This negative predictive value is slightly lower compared to the data in our systematic review of the accuracy of sentinel node biopsy post-neoadjuvant chemotherapy with a pooled negative predictive value rate of 89% (95% CI 85 - 92) [4]. Nathanson *et al*. reported a correlation between the marked nodes (clinical node negative or node positive disease) identified on initial ultrasound of the axilla and sentinel lymph node(s) of 78% [10]. Boughey *et al*. reported that a clip was placed in 170 patients with node positive disease who underwent a sentinel node procedure and axillary lymph node dissection after neoadjuvant chemotherapy [11]. The clip was found as a sentinel node in 107 patients, in the lymph node dissection specimen in 34 patients, and the location of the clipped node was unknown in 29 patients. A combination of sentinel node procedure and a marked node procedure (e.g. radioactive iodine seed, clip placement or preoperative tattooing of the clinical positive-node) in patients with initially clinical node-positive disease might be an interesting new strategy to reduce extend of axillary surgery after neoadjuvant chemotherapy [9, 11, 12]. Boughey *et al.* reported a false negative rate of 6.8% (95% CI 1.9 - 16.5%) in patients with clip placement of the initially clinical node positive disease and a sentinel node procedure after neoadjuvant chemotherapy with removal of at least 2 sentinel nodes followed by axillary dissection [11]. Caudle *et al*. reported in a prospective single-institution study with 208 breast cancer patients a false negative rate of 10.1% (95% CI 4.2 - 19.8) when a sentinel node procedure was done after neoadjuvant chemotherapy, evaluation of the clipped node resulted in a false negative rate of 4.2% (95% CI 1.4 - 9.5) and the combination of sentinel node and a clipped node reduced the false negative rate to 1.4% (95% CI 0.03 - 7.3) [13]. Whether the combination procedures are technically feasible in daily practice and whether the negative predictive failure improves needs to be studied more intensively before implementing this technique in daily practice.

Our findings show that the number of patients requiring axillary treatment is reduced by performing the sentinel procedure post-neoadjuvant chemotherapy in patients with clinically node-negative disease. In a single center study, a total of 3746 patients with clinical T1-T3 node-negative breast cancer underwent a sentinel node procedure from 1994 to 2007, of whom 575 post-neoadjuvant chemotherapy [14]. Sentinel node procedure post-chemotherapy resulted in fewer patients with positive sentinel lymph nodes (absolute reduction of 6.3% for T1, 16% for T2 and 21% for T3 tumors) and decreased the number of axillary dissections in patients with T2 (27% *vs*. 41%, *p* = 0.0001) and T3 tumors (45% *vs*. 66%, *p* = 0.045). There was no difference in regional recurrence rates, after adjusting for clinical stage (0.9% in patients with a sentinel node procedure pre-chemotherapy *versus* 1.2% post-chemotherapy). Recently, a population based study also reported an increased proportion of patients with a negative sentinel node when assessed post- compared to pre-chemotherapy (67% *versus* 54%; *p* = 0.001) [15]. The post-neoadjuvant chemotherapy sentinel node procedure was also associated with significantly less frequent axillary treatment in this study. Reduced axillary treatment may result in less arm and shoulder morbidity as has been reported in the AMAROS trial [16].

A limitation of our and aforementioned studies is that it concerned observational studies. Actually, our study concerned a non-randomized analysis within a randomized frame primarily testing the impact of type of chemotherapy (AC-T *versus* TAC) in the INTENS study and of zoledronic acid in the NEO-ZOTAC study on pathologic complete remission rate. As in- and exclusion criteria of both studies were largely the same, we were able to include a rather uniformly selected patient group for the current research question. Because of the descriptive post-hoc design of the present study we were, however, not able to address the underlying reasons for timing and type of axillary staging in patients with clinically node-negative disease, although we believe we have taken possible confounding factors like tumor size into account by the multivariable analysis. Actually, a trial randomizing between sentinel node procedure pre- *versus* post- neoadjuvant chemotherapy would provide the highest level of evidence. As far as we know, such a trial is not being conducted and will probably not be done in the future, as one may be convinced by the existing evidence from aforementioned observational studies.

It is likely that response to neoadjuvant chemotherapy predicts for the potential benefit of otherwise adjuvant delivered treatment in terms of improved survival. Based on our and other studies, we conclude that in breast cancer patients with clinically node-negative disease the sentinel node procedure is preferentially postponed till after the end of neoadjuvant chemotherapy to take maximum benefit of its effect on nodal down staging. For patients with initially node-positive disease that convert to clinical node-negative disease after neoadjuvant therapy, techniques for more reliable identification of possibly involved nodes needs yet further testing.

## MATERIALS AND METHODS

### Inclusion of patients

Patients were enrolled in two sequentially conducted phase III trials on neoadjuvant chemotherapy under auspices of the Dutch Breast Cancer Research Group (BOOG), the INTENS and the NEOZOTAC study [1, 7]. In the INTENS study, patients were randomized between TAC and AC-T neoadjuvant chemotherapy (doxorubicin (A), cyclophosphamide (C) and docetaxel (T)) [1]. In the NEOZOTAC study, patients were treated with TAC neoadjuvant chemotherapy and randomized between additional zoledronic acid or not [7]. Primary endpoint of both studies was pathologic complete response (pCR). In the INTENS study defined as pCR of the breast and in the NEOZOTAC study as pCR in breast and lymph nodes. All patients provided written informed consent before enrollment and both studies were conducted in accordance with the Declaration of Helsinki and the principles of Good Clinical Practice. Patients were eligible for these studies if clinically positive lymph nodes were present and/or the primary tumor was larger than 3 cm in size in the INTENS study and a minimum of 2 cm in the NEOZOTAC trial. Patients were excluded in the presence of distant metastasis. Of relevance, in the NEOZOTAC study patients with HER2-positive disease were excluded, whereas these could be included in the INTENS study. As a result patients included in NEOZOTAC had on average a more favorable tumor profile: smaller tumors, more node-negative, and more often ER-positive. For the present analysis, patients from both studies were considered eligible, if they had a surgical axillary staging by sentinel node procedure and/or axillary lymph node dissection.

### Axillary staging in INTENS and NEOZOTAC

Baseline clinical nodal status was based on the findings obtained by physical examination and axillary ultrasound, with or without confirmation by fine needle aspiration. In patients with clinically negative lymph nodes a sentinel node procedure could be performed either before start or post-neoadjuvant chemotherapy depending on hospital policy and changing over the years.

In patients with at baseline clinical node-positive disease and a complete response in the axilla post-neoadjuvant chemotherapy (based on physical and ultrasound examination), a sentinel node procedure could be performed as an optional side study post-neoadjuvant chemotherapy, but in this situation always followed by a completion axillary lymph node dissection. In all patients who underwent a sentinel node procedure, dual agent mapping by radio colloid and Patent Blue was used.

### Endpoints

The primary endpoint of the present analysis was the final pathologic node-negative rate in patients with at baseline clinically node-negative disease, that underwent axillary staging by sentinel node biopsy with or without axillary lymph node dissection, in relation to timing pre- or post-neoadjuvant chemotherapy.

Secondary endpoint of the present study was the final pathologic node-negative rate post-neoadjuvant chemotherapy (ypN0) by sentinel node procedure and axillary lymph node dissection in patients with at baseline clinical node-positive disease that converted to clinical node-negative status. Another secondary endpoint was the false-negative rate of the sentinel node procedure in patients with at baseline node-positive lymph nodes and a clinical complete response in the axilla post-neoadjuvant chemotherapy (per protocol followed by an axillary lymph node dissection).

### Definitions

The pathologic nodal status is based on the (combined) results from the sentinel node procedure and results from axillary lymph node dissection whenever performed. Final pathologic node-negative status includes both pN0(i-) and pN0(i+). Pathologic node-positive status includes pN1mi and pN1-3 [17].

The false-negative rate of the sentinel node procedure is obtained by dividing the number of patients who were sentinel node negative but non-sentinel node positive by the number of patients who had a positive sentinel node or non-sentinel node, that is ( 1 - sensitivity). [18]. Non-sentinel nodes were defined as lymph nodes obtained during the completion axillary lymph node dissection.

### Statistics

For clinically node-negative patients at baseline, the effect of the timing of the sentinel node procedure on the combined outcome of sentinel node procedure and axillary lymph node dissection was addressed in a logistic regression model correcting for potentially confounding factors, that is, age at diagnosis, cT-status, histology, tumor grade, hormone receptor status and HER2-status. This yielded the adjusted odds ratio (OR) with 95% confidence interval.
